# Biomimetic Glycosaminoglycan‐Analog Hydrogel for Improved Embolization of Aneurysms: Environment‐Selective Swelling

**DOI:** 10.1002/adhm.202404506

**Published:** 2025-04-07

**Authors:** Sarit S. Sivan, Iris Bonshtein, Maria Khoury, Yevgeniy Kreinin, Dmitry Korneyev, Tirosh Mekler, Sumaya Kaiyal, Iris Sonia Weitz, Netanel Korin

**Affiliations:** ^1^ Department of Biotechnology Engineering Braude College of Engineering Karmiel 2161002 Israel; ^2^ Department of Biomedical Engineering Technion‐Israel Institute of Technology Haifa 32000 Israel

**Keywords:** aneurysm, embolization, injectable hydrogels, minimally invasive therapy, swelling pressure

## Abstract

Injectable hydrogels are promising biomaterials for treating aneurysms, life‐threatening blood‐filled saccular lesions, enabling complete filling of the aneurysm and supporting tissue repair. Yet, the challenge is to enable clinical translation as hydrogels must not protrude into the parent vessel, nor migrate from the aneurysm cavity. Here, injectable, negatively‐charged, biologically and mechanically compatible hydrogels with environment‐sensitive swelling capabilities that cease swelling upon contact with blood are developed. Hydrogels are fabricated by copolymerizing sodium 2‐acrylamido‐2‐methylpropanesulfonic acid (NaAMPS) and 3‐sulfopropyl acrylate (KSPA) by using polyethylene glycol diacrylate (PEGDA). Three formulations (2%, 4%, and 6%) demonstrating a wide range of physiological‐relevant stiffnesses are fabricated. The selected mechano‐compatible 4% hydrogel exhibits a suitable swelling pressure (125 kPa) and supports high endothelial cell viability (> 75%). Importantly, the hydrogel demonstrates a significant differential swell with respect to blood (30 ± 4%), plasma (58 ± 3%), and PBS (82 ± 2%). This environment‐selective swelling, upon exposure to blood, results in minimal directional swelling toward the parent artery, which can improve embolization outcomes. Hydrogel embolization in 3D‐printed aneurysm models subjected to physiological blood flow shows no protrusion toward the main artery while completely blocking flow into the aneurysm. This approach provides promising opportunities for efficient embolization of a variety of aneurysms and vascular malformations.

## Graphical abstract

Biomimetic glycosaminoglycan analog hydrogels exhibit suitable and robust mechanical properties, as well as hemocompatibility and biocompatibility, making them ideal for the embolization of intracranial aneurysms. Notably, the hydrogel's environment‐selective swelling properties enable complete blockage of the aneurysm cavity while preventing protrusion into the main vessel and ensuring better localization within the aneurysm cavity.

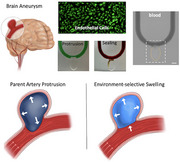

## 1 Introduction

Intracranial aneurysms are life‐threatening blood‐filled saccular lesions located on cerebral arteries, affecting ≈2–6% of the population.^[^ ^1^ ^]^ While most aneurysms remain asymptomatic, when a cerebral aneurysm ruptures, the mortality rate is high, and survivors often suffer long‐term disabilities.^[^ ^2^, ^3^ ^]^ Current advanced approaches for the treatment of high‐risk aneurysms are based on minimally invasive intravascular procedures, mainly coiling and stenting which aim to seal the aneurysm, detaching it from blood flow. However, despite advancements in endovascular treatment approaches, the use of metallic implants to embolize aneurysms is not ideal, as they do not completely fill the aneurysm cavity, can cause damage, may require anticoagulant medication, and are not fully biocompatible.^[^ ^4^ ^]^ Additionally, some aneurysms are difficult to treat with coils because of their irregular shape. Thus, there is a clinical need for effective and safe localized solutions for the treatment of aneurysms.

**Figure 1:**
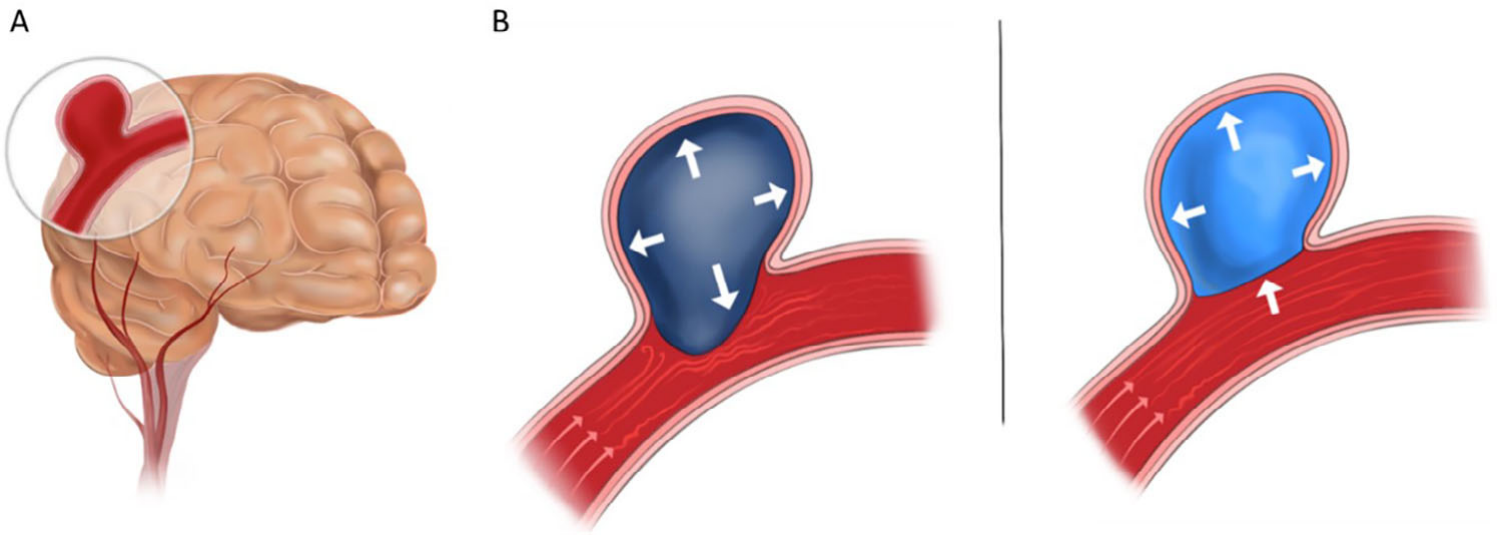
An illustration presenting embolization of an aneurysm using a hydrogel that upon swelling protrudes toward the parent artery blood vessel and our suggested environment‐sensitive hydrogel‐based strategy to overcome this issue. A) An illustration of a saccular intracranial aneurysm. B) (left) Iso‐directional swelling of embolic hydrogels can result in protrusion into the parent artery. (right) a GAG analog embolic hydrogel exhibiting selective swelling–, i.e., minimal swelling toward the neck–can prevent protrusion into the parent artery and promote better localization of the hydrogel within the aneurysm cavity.

Recently, with advancements in biomaterial engineering, the study of biocompatible liquid embolic agents gained renewed interest due to their potential to completely fill complex aneurysm geometries as well as provide suitable scaffolds for vessel repair. In this context, hydrogels are ideal for aneurysm treatment as they mimic many properties of the native tissue and promote cell and tissue growth; and yet, their properties need to be tailored to properly address this challenge.^[^ ^5^, ^6^ ^]^ As part of these advancements, an alginate injectable hydrogel formulation, EmboGel, has been developed and commercialized as an embolization material that solidifies via calcium chloride‐induced polymerization.^[^ ^7^, ^8^ ^]^ However, the main challenge in using such hydrogels for aneurysm embolization is that they can partially reflux into the parent artery, thus interfering with blood flow.^[^ ^9^ ^]^ More generally, a variety of injectable hydrogel‐based embolization materials with various modes of solidification have been investigated, including thermo‐responsive hydrogels,^[^ ^10^ ^]^ shear‐thinning biomaterials,^[^ ^11^, ^12^ ^]^ pH‐responsive gels,^[^ ^13^ ^]^ and photo‐crosslinked hydrogels.^[^ ^14^, ^15^ ^]^ Although these and other new injectable biomaterials have shown promising results as embolic materials for a variety of applications, such as embolizing Arteriovenous Malformations (AVMs) and both malignant and benign tumors,^[^ ^12^, ^16^ ^]^ the case of aneurysm embolization is more complex and challenging. To prompt successful embolization of aneurysms, hydrogels should be injectable, biocompatible, fill the entire aneurysm sac, solidify within a few minutes, possess suitable mechanical properties, and also serve as a favorable substrate for endothelial growth. Furthermore, to enable clinical translation, the hydrogels must not protrude into the parent vessel nor migrate from the aneurysm cavity–a major challenge that exists in all currently examined embolic hydrogels, which hinders their specific application for the treatment of aneurysms. Although hydrogels can naturally swell, expanding their volume and applying swelling pressure on their surrounding environment, which can contribute to their localization inside the aneurysm cavity, their swelling can also lead to protrusion toward the parent artery (see **Figure** **1**). For this reason, so far, hydrogels for aneurysm embolization were designed to exhibit minimal swelling.^[^ ^9^ ^]^ Thus, an embolic hydrogel that can exhibit selective swelling, i.e., swelling toward the aneurysm wall with minimal swelling toward the neck and the parent artery, can provide improved aneurysm embolization and treatment, see Figure 1B.

We have developed a novel series of osmo‐responsive biomimetic glycosaminoglycan (GAG) analogs based on sulfonate‐containing precursor monomers for the regeneration and repair of load‐bearing tissues.^[^ ^17^, ^18^ ^]^ As the key component that controls osmolarity and hydration in load‐bearing tissue (e.g., intervertebral disc and cartilage) is the negatively charged sulfated glycosaminoglycan (sGAG) chains,^[^ ^19^ ^]^ the sulfonate group was viewed as an effective surrogate for the naturally occurring sulfates in the GAG chains. These GAG analogs provide an intrinsic swelling pressure,^[^ ^17^ ^]^ which can be further manipulated to meet the requirements occurring in various load‐bearing environments. Additionally, these GAG analogs are suitable to promote cell growth, and their swelling and mechanical properties can be tuned for various applications.

This study presents the use of GAG analogs as potential embolic agents for a stable occlusion of side aneurysms, while ensuring no protrusion into the parent vessel owing to their minimal swelling at the aneurysm neck, when in contact with blood. To this aim, GAG analogs of various crosslinking densities have been studied for their mechanical properties and biocompatibility under well‐controlled settings, and their ability to swell in the presence of different biofluids was studied in vitro. Following this and based on the hydrogel's selective swelling, successful embolization in 3D printed aneurysm models subjected to a physiological blood flow regime was demonstrated. The results show that no protrusion toward the main artery occurs, while the hydrogel in the cavity completely blocks the flow into the aneurysm sac.

## 2 Results and Discussion

### 2.1 Synthesis and Mechanical Characterization of GAG Analog Hydrogels

GAG analog hydrogels were prepared using sulfonate‐containing monomers polymerized in the presence of a crosslinking agent via redox polymerization at several concentrations using typical two‐part pre‐gel components, mixed in equal volumes.

**Figure 2:**
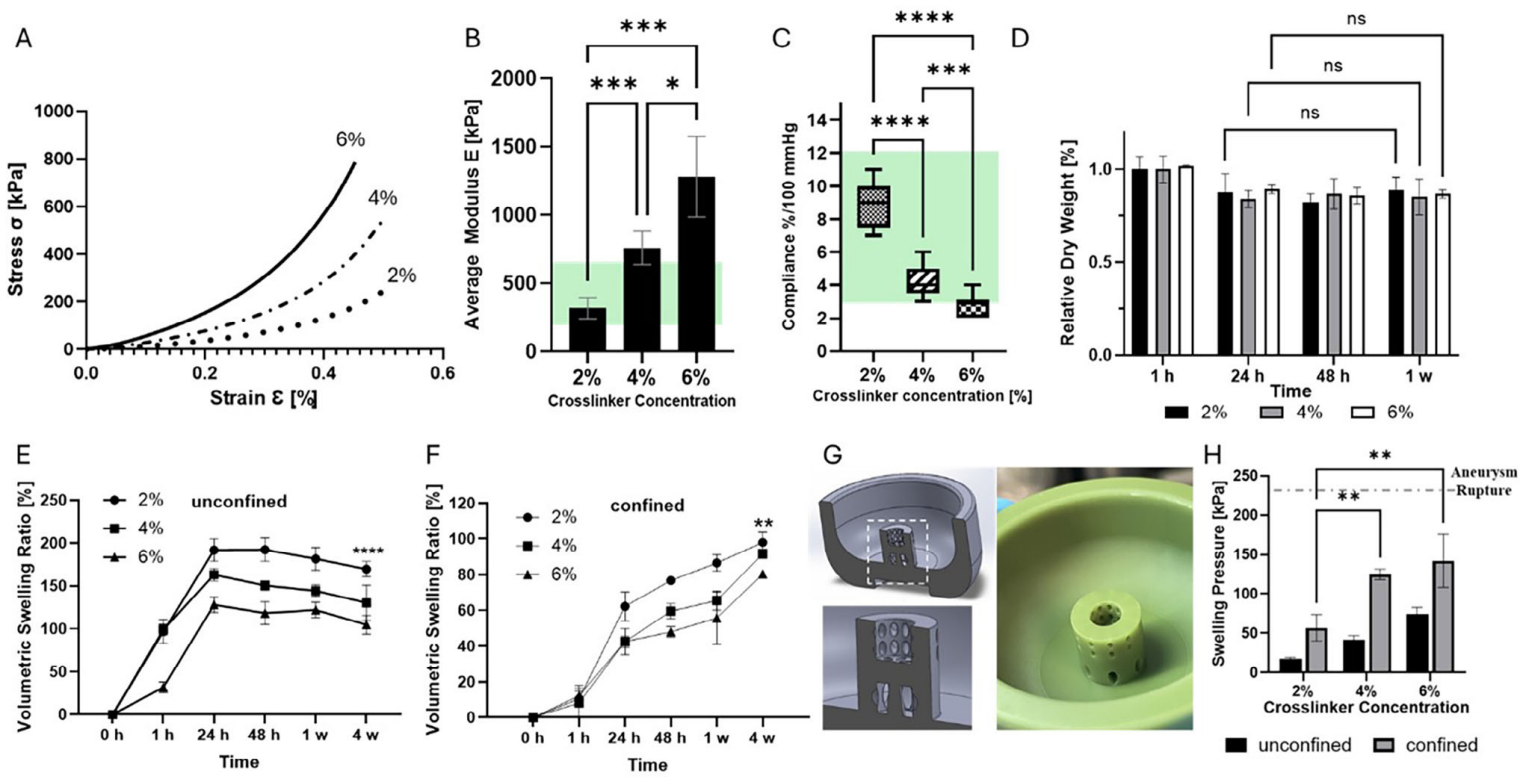
Mechanical and swelling properties of GAG analog hydrogels with different crosslinking densities A) Strain–stress curve performed 1 h after mixing the precursor monomers using different crosslinker concentrations (2%, 4%, and 6%); B) Compressive elastic modulus of the hydrogels; the green range between 200 and 600 kPa, represents the elastic modulus of the human brain aneurysms under strains lower than 50%; C) Compliance of the hydrogels under physiological pressures (80–120 mmHg); the green range of 3–12%/100 mmHg, represents the compliance of the native human arteries; D) Volumetric swelling of GAG analog hydrogels crosslinked at 2%, 4%, and 6% in PBS over 4 weeks under unconfined setting; E) Relative dry weight measurements of GAG analog hydrogels over a period of 4 weeks under unconfined setting. F) Volumetric swelling of GAG analog hydrogels crosslinked at 2%, 4%, and 6% in PBS over 4 weeks under confined geometry; G) Computer‐Aided Design model and its corresponding 3D printed confinement device, where the hydrogel was placed to mimic the confined conditions residing in the aneurysm cavity; H) Swelling pressure as a function of crosslinking density under confined and unconfined settings (*p* < 0.01).

The mechanical properties of GAG analog hydrogels were tested and compared to the requirements for embolizing aneurysms.^[^ ^9^, ^20^ ^]^ Hydrogels were prepared at three crosslinking densities (2%, 4%, and 6%) and subjected to compression tests. Data are presented in Figure. 2 and **Table** **1**. As the crosslinking density increased, the hydrogels became stiffer, as presented by the strain‐stress curve (**Figure** **2**A) and by the elastic moduli (Figure 2B). The elastic moduli obtained were 319 ± 71, 759 ± 112, and 1282±271 kPa for the 2%, 4%, and 6% hydrogels respectively (Figure 2B). These values are comparable with the elastic modulus range of human brain aneurysms, reported to be between 200 and 600 kPa under strains lower than 50%.^[^ ^21^ ^]^ These values are higher for healthy vessels.

**Table 1:** Failure stress and strain for GAG analogs as a function of crosslinking density.

	Failure Stress and Strain		
	2%	4%	6%
**Stress [kPa]**	686 ± 77	890 ± 255	752 ± 68
**Strain [%]**	64 ± 2	58 ± 6	45 ± 2

Another important property, that dictates blood vessels’ ability to deform under physiological conditions, is their compliance under physiological pressures. The compliance values found were 8.7 ± 1.3, 4.5 ± 1.0, and 2.8 ± 0.7%/100 mmHg for the 2%, 4%, and 6% hydrogels respectively (Figure 2C). These values are comparable with the compliance of the native human arteries, which is in the range of 3–12%/100 mmHg.^[^ ^22^ ^]^ Thus, the 2% and 4% hydrogels demonstrate elastic moduli similar to the native vessel, whereas the 4% and 6% hydrogels have mechanical compliance similar to the native vessel.

As presented in Table 1, the strain breakup of the hydrogels is shown to be inversely proportional to the elastic modulus. Thus, the softer 2% hydrogel exhibited the highest yield strain compared to the other hydrogels (690 ± 70, 890 ± 255, and 750 ± 70% for the 2%, 4%, and 6% respectively). All the tested hydrogels showed failure stress above 100–150 kPa, which is the circumferential stress applied by the blood pressure.^[^ ^23^ ^]^ Interestingly, as shown in Table 1, the 4% hydrogel exhibited the highest failure stress compared to the 2% and 6% hydrogels. Although, as expected, the 6% hydrogel has the highest elastic modulus, it is more brittle and its breakup strain is significantly lower than the 4% (58 ± 6% for the 4% compared to 45 ± 2 for the 6% hydrogel, Table 1); this lower failure strain results with the 4% having the highest failure stress. Taken altogether, the 4% hydrogel shows mechanical properties compatible with native arteries.

### 2.2 Degradation, Hydration, and Swelling Pressure

Measurements of the dry weight of GAG hydrogels over time were performed for a period of 4 weeks (Figure 2D). Our results demonstrate the stability of the GAG‐analog hydrogels over time. Volumetric swelling of GAG analogs was tested under confined and unconfined configurations. When tested under the unconfined settings, the volumetric swelling ratio of all hydrogels increased with crosslinker density until equilibrium was reached after 24 h, at which time the volumetric swelling ratio remained stable for at least 4 weeks: 192 ± 13%, 163 ± 7%, and 128 ± 9% for the 2%, 4%, and 6% respectively (Figure 2E).

Swelling under confined settings (inside Eppendorf tubes) mimicking native aneurysm conditions showed an increase with crosslinking density; however, to a lesser degree compared to the unconfined conditions: 98 ± 5, 92 ± 3, and 80 ± 2% for the 2%, 4%, and 6% respectively (Figure 2F), and to 90–100% swell for all hydrogels after 4 weeks under confined setting.

Additionally, swelling pressure measurements were performed under confined conditions and compared to the physiological value for aneurysm rupture.^[^ ^9^ ^]^ The hydrogels were placed in a custom‐made device designed to mimic a fully confined hydrogel, representing a more constrained condition compared to hydrogels placed in an aneurysm with an opening in its neck (Figure 2G). The results show that the swelling pressure increases significantly (*p* < 0.01) with crosslinking density. The maximal value obtained for the 6% hydrogel, under this condition, was 142 ± 33 kPa; which is lower than the physiological rupture values of 230 ± 60 kPa^[^ ^24^ ^]^ (no statistical significance between the 6% and the physiological value). This emphasizes the fact that the 4% hydrogel has improved mechanical properties compared to native arteries (Figure 2H). In practice, the swelling pressure might therefore be lower in semi‐confined conditions similar to those residing inside an aneurysm. Additionally, results by others testing a shape memory foam that did not induce critical circumferential stresses support the idea that even higher pressures than 230 ± 60 kPa can be sustained.^[^ ^25^ ^]^

### 2.3 Biocompatibility

**Figure 3:**
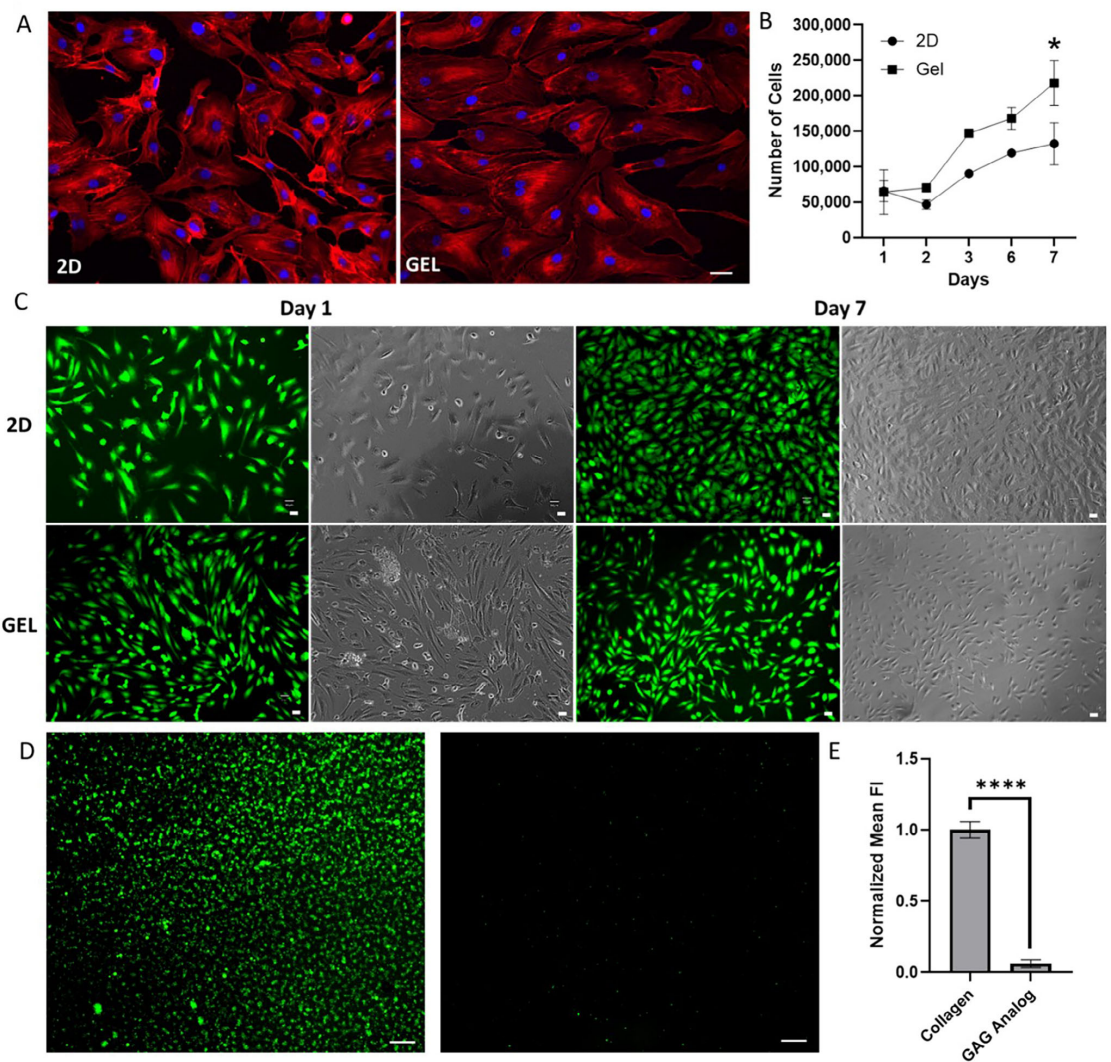
Biocompatibility of HUVECs grown on 4% GAG analog hydrogels. A) HUVECs were grown on 4% GAG analog hydrogels (left) and on 2D plates (right) and cells were stained 2 days post‐seeding, using Phalloidin and DAPI. Scale bar: 10 µm. B) Cell viability of HUVECs grown on 4% GAG analog hydrogel and 2D plates was quantified over 7 days using the Alamar Blue assay. C) Cell viability of HUVECs (50 000 cells were initially seeded) grown on 4% GAG analog hydrogel and 2D plates were imaged at 1‐ and 7‐days following seeding, using the Ethidium Homodimer I and Fluorescein Diacetate. Respective phase contrast images are also presented. Scale bar: 50 µm. D) Fluorescently stained platelets adhered to collagen (left) compared to GAG analog hydrogel (right), where minimal platelets adhesion was observed. Scale bar: 100 µm. E) Quantification of the fluorescent signal obtained from platelet adhesion images, comparing the normalized mean fluorescence intensity (FI) of collagen and the GAG analog hydrogel (*p* < 0.0001).

As mentioned earlier, the 4% hydrogel shows mechanical properties compatible with the native artery. Therefore, the capability of the 4% hydrogel to support the growth of endothelial cells, lining all blood vessels, was tested. To this aim, human umbilical vein endothelial cells (HUVECs) were cultured on fibronectin‐coated 4% hydrogels and tested for their viability and morphology after 48 h (**Figure** **3**). The general morphology and actin filaments, as demonstrated by the dual staining of Phalloidin (in red) and DAPI, showed no difference between cells grown on the hydrogel and those grown on 2D plates (Figure 3A). The viability of cells grown on GAG analog hydrogels and on 2D plates was quantified using the Alamar Blue assay and qualitatively examined using the ethidium homodimer I and Fluorescein Diacetate staining followed by fluorescent microscopy imaging (Figure 3B,C). Cells grown on the hydrogel proliferated more rapidly than cells grown on 2D plates (growth rate of 70% compared to 50%, *p* < 0.05), demonstrating the hydrogel's capability to serve as a suitable substrate for endothelial cell re‐growth. Moreover, qualitative evaluation of the viability after 1 and 7 days, using the ethidium homodimer I and Fluorescein Diacetate staining and phase contrast imaging, showed that cells were viable both on 2D plates and on the hydrogel for at least 7 days (viability > 98%) (Figure 3C; Figure S1, Supporting Information). In our previous study,^[^ ^18^ ^]^ the toxicity of GAG analogs (1%) and of its individual components was tested on fibroblasts (L929) and bovine nucleus pulposus disc cells. The viability of cells after 24 h exposure to the major components, NaAMPS and KSPA, in concentrations similar to the typical residual monomer content (0.4 ± 0.04%, v/w) was not reduced, in line with toxicological studies previously reported for these monomers.^[^ ^26^, ^27^, ^28^, ^29^ ^]^ It is worth noting that according to ISO 10993–4, the required viability needs to be higher than 70%.^[^ ^30^ ^]^ In our case, cell viability is higher, and also cells proliferate at a similar or even a higher rate when compared to tissue culture plates.

To evaluate hemocompatibility of GAG analog hydrogel, hemolysis and platelets adhesion assays were performed. Our results showed that hemolysis rate (%HR) was 3.4 ± 1.1%, below the international standard of biomaterials hemolysis rate threshold of 5%, indicating that the GAG analog hydrogel has good blood compatibility.^[^ ^31^ ^]^ Furthermore, platelets adhesion to the GAG analog hydrogel was minimal, especially when compared to adhesion on collagen (< 7%, *p* < 0.0001). These findings suggest that the GAG analog hydrogel is generally hemocompatible and does not induce platelets aggregation. However, further testing is needed to thoroughly assess the long‐term hemocompatibility of the hydrogels.

It is worth noting that some of the hydrogels explored for aneurysm embolization, including alginate, exhibit significantly lower cell viability compared to our GAG analog hydrogel formulation, potentially limiting vessel healing.^[^ ^5^ ^]^

### 2.4 Hydrogel Swelling and Evaluation of Hydrogel‐Blood Interactions by Attenuated Total Reflectance‐Fourier Transform Infrared (ATR‐FTIR(Spectroscopy

**Figure 4:**
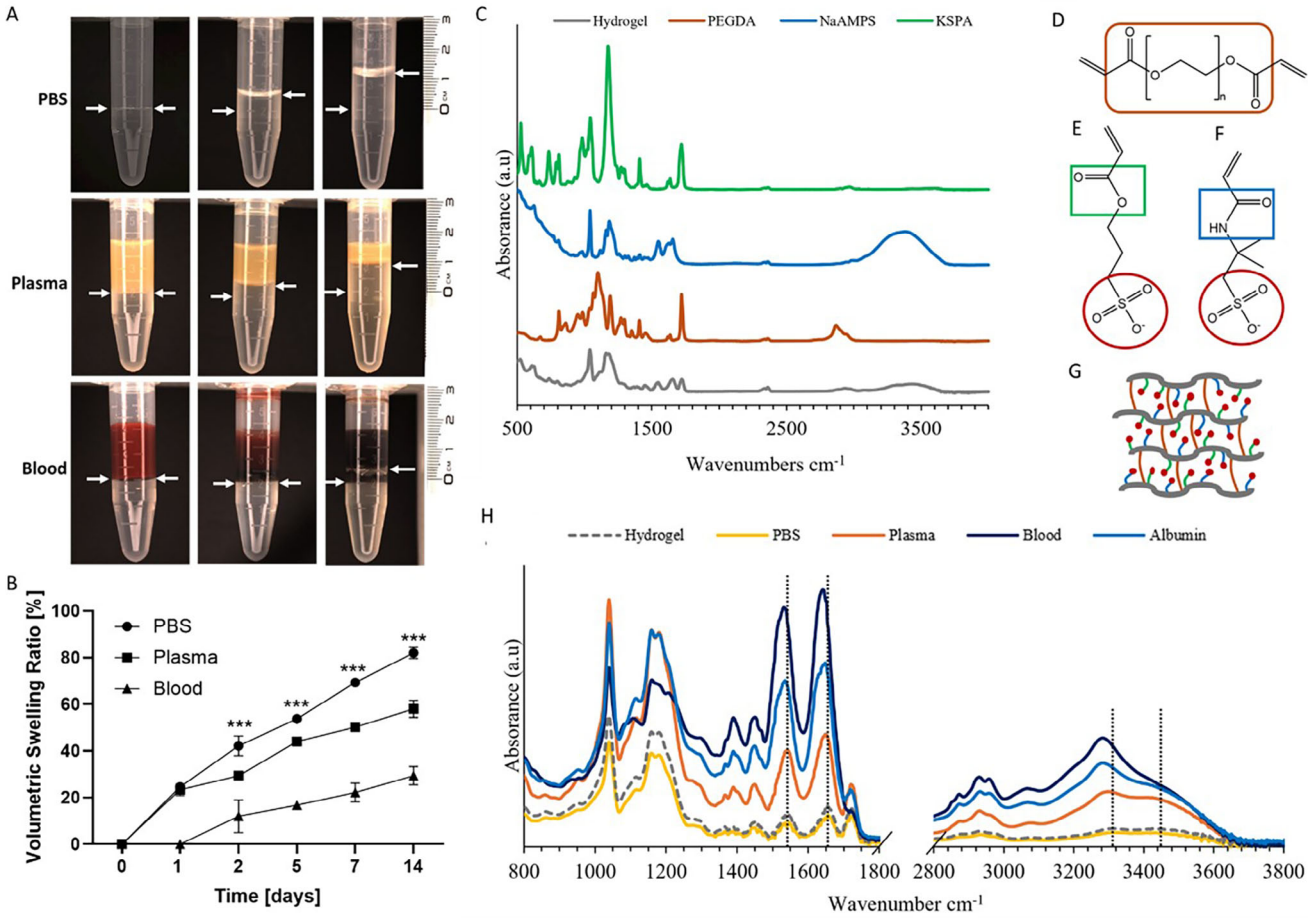
Swelling of 4% GAG analog hydrogels in the presence of human blood, plasma, and PBS under static conditions and respective ATR‐FTIR characterization. A) Swelling experiments of 4% hydrogels in 5 mL Eppendorf tubes following incubation with different fluids over a period of 14 days. Left arrows indicate the gel at t = 0 and the right arrows indicate the end of the experiment; B) Respective volumetric swelling ratios measurements; C) Spectra of GAG analog hydrogel and its building units: D) PEGDA (crosslinker), E) KSPA, and F) NaAMPS monomers; G) Schematic illustration of the hydrogel network and H) Spectra of the hydrogel after treatment with blood, plasma, albumin, and PBS, compared to the untreated hydrogel. The dashed lines indicate redshifts in amide bond vibrations compared to the untreated hydrogel.

To study hydrogel swelling under static conditions and a confined setting, cured hydrogels were allowed to swell in Eppendorf tubes, in the presence of PBS, plasma, and human blood for two weeks and the relative change in volume was measured (**Figure** **4**A). In the presence of PBS, the volumetric swelling ratio was the highest, 82 ± 2%, compared to the significantly lower value obtained in the presence of blood 30 ± 4% (*p* < 0.001), Figure 4B.

To explore the interactions at the interface between the hydrogel and the blood, the FTIR spectra were studied. FTIR spectra of the two monomers, KSPA and NaAMPS, the crosslinker PEGDA, and the cured GAG analog hydrogel are shown in Figure 4C–G. After the hydrogel is formed, the C═C stretching vibration at ≈1625–1635 cm^−1^ and the out‐of‐plane C─H bending vibration at 983–985 cm^−1^ disappear, indicating that copolymerization proceeded through carbon‐carbon double bond opening.^[^ ^32^ ^]^ The hydrogel spectrum showed the characteristic peaks from KSPA, NaAMPS, and PEGDA. The symmetric and antisymmetric S═O stretching vibrations of the SO_3_^−^ anionic group of KSPA and NaAMPS appeared at 1039 and 1181 cm^−1^, respectively.^[^ ^33^, ^34^ ^]^ The carbonyl absorption of the ester group of KSPA and PEGDA was observed at 1720 cm^−1^.^[^ ^35^, ^36^ ^]^ The remaining ester‐related vibrations, including the C─C(═O)−O stretch (1160‐1210 cm^−1^), and the O−C─C stretch (1030–1100 cm^−1^),^[^ ^37^ ^]^ were likely overlapped by the S═O absorption. In addition, the hydrogel spectrum showed the characteristic amide groups from NaAMPS: amide I (1655 cm^−1^, attributed to C═O stretching with minor contribution from C─N stretching), amide II (1546 cm^−1^, resulting from N─H bending and C─N stretching), and amide A (broad peak ≈3400 cm^−1^, arising from N─H stretching)^[^ ^34^, ^38^ ^]^

GAG analog hydrogels were incubated for 24 h with fresh human blood, plasma, and PBS at 37 °C, and their ATR‐FTIR spectra were analyzed. The resulting spectra are shown in Figure 4H. The spectra of treated hydrogels closely resembled that of the untreated hydrogel, showing characteristic typical bands. However, it can be noticed that following treatment with blood, amide vibration exhibited redshift, while other bands, such as the stretching vibrations of the S═O remained unchanged. This red‐shift effect was also observed in the hydrogels treated with plasma and albumin but was absent after PBS treatment. These findings suggest that albumin is likely to be a significant contributor to the reduction in water uptake capacity, which subsequently affects the hydrogel's swelling behavior (Figure 4B). Albumin is the most abundant protein in plasma, characterized by an anionic nature, and plays a critical role in maintaining osmotic pressure.^[^ ^39^ ^]^ Therefore, we propose the following interactions that albumin may induce at the interface with the hydrogel.

i) Repulsive interactions between the negatively charged albumin (under physiological conditions) and the anionic sulfonate groups of the hydrogel. Hence, the conformation of the side chains of the NaAMPS and KSPA moieties changes, with the SO_3_^−^ groups facing inward and the amide/ester groups facing outward. ii) Attractive interactions between the amino acid side chains of albumin and the amide bond of the NaAMPS side chains. As seen in the spectra (Figure 4G), when the hydrogel was treated with blood, plasma, and albumin, the amide I band shifted from 1655 cm^−1^ to 1644, 1648, and 1648 cm^−1^, while amide II shifted from 1546 cm^−1^ to 1537, 1542, and 1535 cm^−1^, respectively. These redshifts indicate bond lengthening due to enhanced hydrogen bonding. Moreover, the broad N─H band centered at ≈3400 cm^−1^ became more intense, red‐shifted, and appeared as an unsymmetrical doublet, implying rotational conformations (cis‐trans) within the N─H stretching band.^[^ ^40^ ^]^ These combined interactions likely influence the hydrogel's structure, reducing the accessibility of the SO₃⁻ groups to water molecules, thereby hindering hydrogel swelling. Albumin is likely a key contributor influencing the swelling behavior of the hydrogel. However, given the differences in swelling behavior between blood and plasma, the full mechanism requires further investigation.

### 2.5 GAG Hydrogel Aneurysm Embolization: Environmental‐Dependent Swelling

First, the injectability of the pre‐gel solutions was studied to ensure their suitability for future catheter‐based treatments. To this aim, viscosities of the pre‐gel solutions were measured. For all formulations tested, the viscosity was found to be less than 10 mPa s (**Table** **2**), which is lower than that of currently used liquid embolic agents (e.g., Onyx‐ 25 mPa∙s and PHIL–80 mPa s),^[^ ^16^ ^]^ thus demonstrating suitability for intravascular catheter‐based injections.

**Table 2:** Viscosity and density of the pre‐gel solutions used for the preparation of GAG analogs.

Property	Part (a)	Part (b) (2%)	Part (b) (4%)	Part (b) (6%)
**ρ (g mL^−1^)**	1.14	1.19	1.16	1.16
**η [mPa**∙**s]**	1.71 ± 0.01	4.37 ± 0.05	4.95 ± 0.05	6.84 ± 0.43

Parts (a) and (b) solutions are described in the Experimental Section.

**Figure 5:**
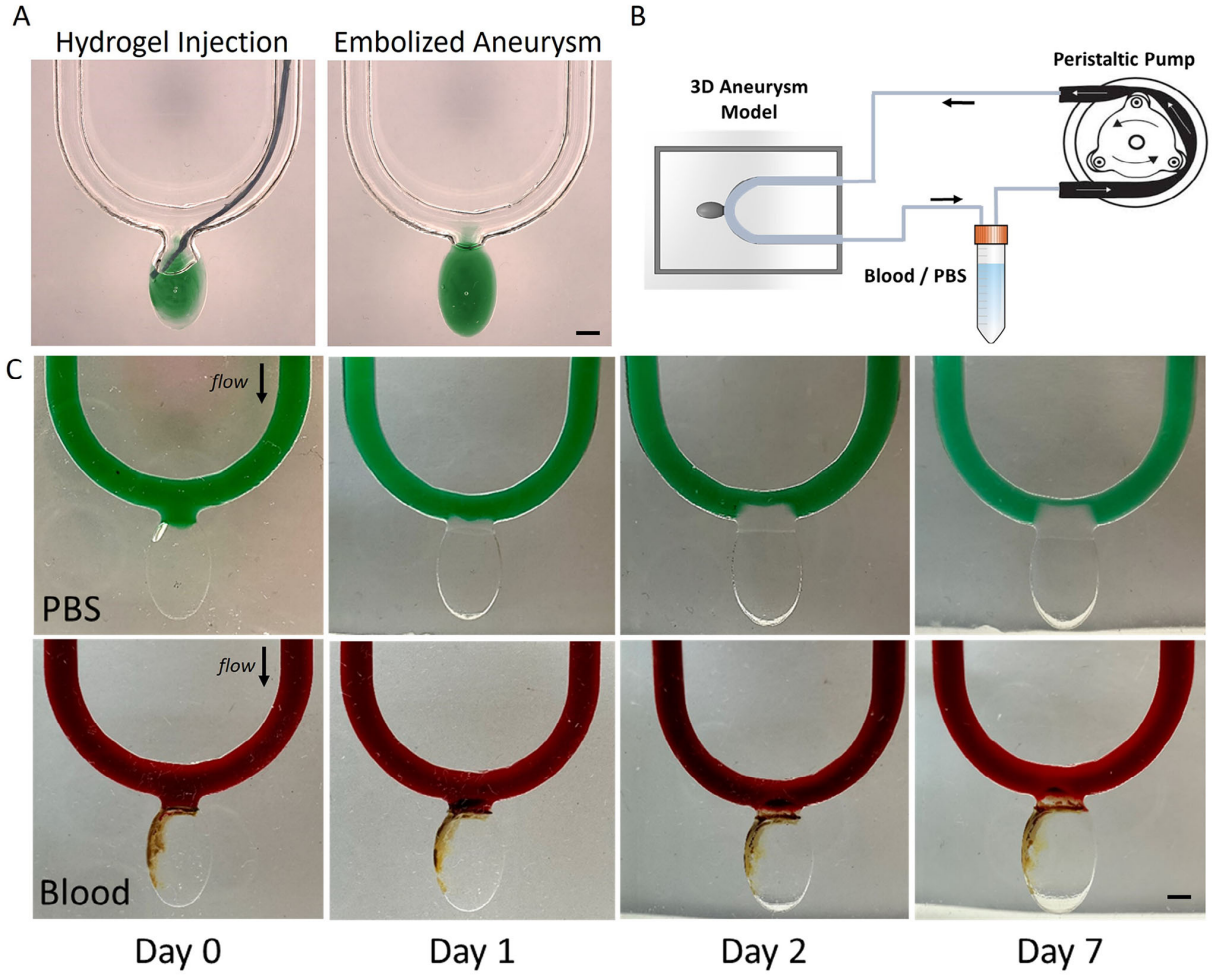
Aneurysm embolization using GAG analog hydrogels. A) Aneurysm filling is performed using a catheter to inject the precursor monomers until complete filling of the aneurysm cavity is reached. Scale bar: 3 mm; B) Following hydrogel formation, the embolized model is connected to a perfusion system comprised of a peristaltic pump through which fluids (PBS/blood) are perfused in a closed‐circuit; C) Time‐lapse imaging of swelling experiments in 3D‐printed aneurysm models subjected to flow, showing hydrogels swell in the presence of PBS (top) and blood (bottom) over a period of 7 days. Scale bar: 1.5 mm.

To examine the ability to form GAG hydrogels in an aneurysm cavity via intravascular catheter‐based infusion, experiments were performed in 3D transparent casted aneurysm models. To solidify the hydrogel, the two pre‐gel solutions were infused through separate tubes that merged into a mixing reservoir at the tip. At the end of this tip, the mixed solutions were infused into the cavity, resulting in a hydrogel that filled the aneurysm (**Figure** **5**A). Injection of two‐component hydrogels for embolizing cerebral aneurysms can be achieved using conventional dual‐catheter delivery systems (Figure S2, Supporting Information). An example of a co‐injection system is used in a cross‐linked ECM‐derived matrix for the treatment of cerebral aneurysms, where gelatin‐thiol (Gel‐SH) and hyaluronic acid vinyl sulfone (HA‐VS) are combined.^[^ ^41^ ^]^ Additionally, the embolic agent EmboGel, a blend of iohexol and alginate, forms a hydrocoil that is delivered through a coaxial catheter with a distal mixing tip. In this system, the alginate is mixed with a calcium chloride solution, thus initiating polymerization.^[^ ^8^ ^]^ In our system, the two hydrogel components are injected using two separate catheters, which converge into one tubing where they mix and interact, before filling the aneurysm. Then, the hydrogel mixture is injected from the tubing into the aneurysm where it solidifies to embolize the aneurysm. In future developments, a dual catheter specifically designed for GAG analog hydrogels, will be needed to streamline this process. To test the effect of swelling in realistic aneurysm geometries, swelling experiments were also conducted in 3D aneurysm models connected to a closed‐circuit perfusion system (Figure 5B), where the overflow toward the parent artery was also monitored, upon interacting with different fluid environments, over a period of 7 days. Here too, in the presence of PBS, the hydrogels expanded significantly and protruded into the lumen of the parent artery, whereas in the presence of blood, hydrogels remained confined within the aneurysm cavity (Figure 5C).

Furthermore, it is important to note that in a clinical setting, complete aneurysm filling can be monitored through imaging during the procedure by confirming that a radiolabeled embolic agent fully occupies the cavity. Since aneurysms vary in shape and size, using a smaller amount of hydrogel to account for swelling and achieve complete filling requires accurately determining the exact volume of the cavity in advance. This process also relies on the hydrogel swelling behavior, which may deviate from its expected value, adding complexity to the embolization procedure and increasing the risk of critical errors. For this reason, hydrogel embolic agents for aneurysm treatment have so far been designed to exhibit minimal swelling (< 25%).^[^ ^9^ ^]^ Our proposed GAG analog hydrogel, however, allows for selective swelling, enabling the use of a high‐swelling hydrogel while ensuring that, once fully deployed, it does not protrude into the parent artery.

### 2.6 Long‐Term Embolization Under Flow

**Figure 6:**
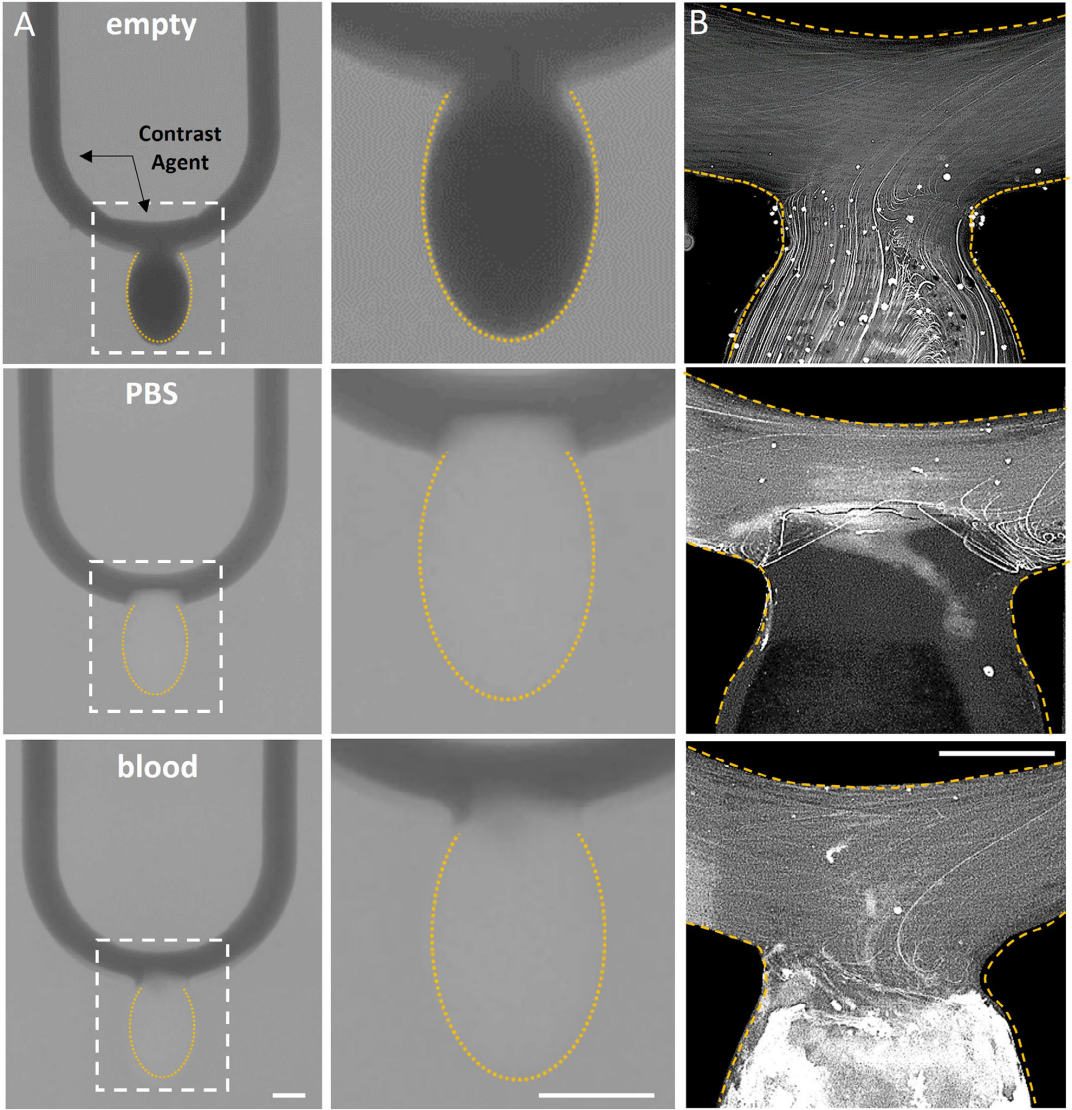
Unidirectional swelling of GAG analog hydrogels in a 3D printed aneurysm model under flow. A) CT images of 3D aneurysm model, either empty (no hydrogel, top) or embolized with 4% hydrogels in the presence of PBS (middle) or the presence of human blood (bottom), all under physiological flow. Scale bar (right and middle columns): 3 mm; B) Respective pathlines of 10 µm fluorescently labeled polystyrene particles under physiological flow. Scale bar: 1.5 mm.

The ability of GAG analogs to embolize an aneurysm over time, while ensuring proper sealing of the cavity without protrusion into the parent artery, was tested under flow using a 3D aneurysm model. GAG analogs were first cured within the aneurysm cavity, followed by perfusion of blood or PBS over a period of 2 weeks. Time‐lapse imaging was performed to evaluate the hydrogel's capability to seal the aneurysm without any protrusion toward the main artery. The effectiveness of the aneurysm neck sealing was further demonstrated using microCT imaging (**Figure** **6**A) and particle pathline experiments (Figure 6B). The efficacy of the embolization was validated by tracking the pathlines of flowing fluorescent particles. Prior to treatment, recirculating flow patterns were observed within the aneurysm cavity. After embolization with the GAG analog hydrogel, these flow patterns were no longer detected within the cavity, and the pathlines were confined to the parent artery region. This demonstrated the blockage of flow and kinetic energy within the aneurysm, a parameter that is known to correlate with effective aneurysm treatment.^[^ ^42^ ^]^

Results from both images show minimal (if any) contrast agent and particle pathlines within the cavity area compared to the empty aneurysm, thus demonstrating complete blockage of blood flow within the cavity.

The GAG analog hydrogel studied here, similar to other hydrogel embolic agents,^[^ ^5^ ^]^ can tune its mechanical properties by adjusting factors such as monomer concentration, monomer molecular weight and crosslinking ratio. Poupart et al.^[^ ^9^ ^]^ developed a photopolymerizable poly(ethylene glycol) dimethacrylate (PEGDMA) hydrogel with optimal mechanical properties for brain aneurysm embolization, as demonstrated in their comprehensive study. Similarly, we have optimized our hydrogel formulation to achieve comparable mechanical characteristics. However, in contrast to our hydrogel, the PEGDMA hydrogel was designed to minimize swelling (< 25%) and prevent invasion into the parent vessel, while our GAG analog hydrogel exhibits selective swelling. This allows it to expand toward the cavity walls (> 80% swelling in PBS) without protruding into the parent artery. In addition, the PEGDMA hydrogel relies on photopolymerization for crosslinking, a method that requires further development.^[^ ^15^ ^]^ Other hydrogel formulations investigated for aneurysm embolization, such as alginate‐based hydrogels exhibit weaker mechanical properties that may lead to fracture and partial reflux into the parent artery, as observed in preclinical models^[^ ^43^ ^]^ and require further modification such as double‐crosslinked alginate‐based hydrogel microfibers.^[^ ^44^ ^]^

## 3 Conclusion

In summary, our study introduces a new method for efficient embolization of aneurysms, based on hydrogels with environmental‐dependent swelling capabilities. In our case, negatively charged GAG analogs showed minimal (if any) swelling upon contact with blood, thus avoiding hydrogel penetration into the parent artery while allowing swelling toward the aneurysm walls to potentially improve its localization within the aneurysm cavity. The GAG analog hydrogels demonstrated suitable and robust mechanical properties as compared to native arteries under physiological pressure. In addition, with regards to biocompatibility, the GAG analog hydrogel allowed the growth of endothelial cells, which line blood vessels.

Our flow experiments show that the cured hydrogels completely blocked the aneurysm cavity, as demonstrated by the absence of pathlines within the aneurysm cavity. Moreover, when blood was circulated, no protrusion toward the main vessel was observed.

This, together with their precursor injectability, suggests that these environmentally dependent hydrogels hold potential for blood vessel and aneurysm embolization of complex geometries as well as provide a suitable scaffold for vessel repair. It is worth noting that although this work focused on GAG analogs, other negatively charged hydrogels can be designed to show such environmental selective swelling that can be beneficial for embolization. The experimental procedures outlined in this work provide a reliable tool for identifying other gel types with suitable environmental selective swelling properties for use in aneurysm treatment. Additionally, our preliminary data show that other crosslinkers can be used, e.g., alcian blue, to facilitate the cross‐linking kinetics. In this work, we focused on a proof‐of‐concept study; however, future work should test the potential of these hydrogels for other applications and adjust their composition and properties to treat various aneurysms and meet other medical challenges. Long‐term studies should consider the safety, efficacy, and long‐term effectiveness of these hydrogels in animal models. Additionally, a proper delivery catheter system needs to be designed to meet clinical and regulatory requirements. Moreover, filling aneurysms remains highly user‐dependent, and advances are needed to guide the filling and ensure it is performed accurately. Overall, the proposed injectable, environmentally dependent hydrogels serve as potentially effective embolic agents for the treatment of aneurysms and other vascular malformations.

## 4 Experimental Section

### Materials

ECM Medium, FBS, ECGS, and penicillin‐streptomycin solution were purchased from ScienCell, California, USA. Trypsin EDTA Solution B was from Biological Industries (BI), Beit HaEmek, Israel. Sodium 2‐acrylamido 2‐methyl propane sulfonic acid (NaAMPS) (58% in aqueous solution), polyethylene glycol diacrylate (PEG575DA, Mn ≈500), ascorbic acid (AA), potassium peroxymonosulfate (Oxone), 3‐Sulfopropyl acrylate potassium salt (KSPA), fibronectin, ethidium homodimer, and DAPI were purchased from Sigma, Rehovot, Israel. Phalloidin red was from Invitrogen, ThermoFisher Scientific, UK. Fluorescein Diacetate from Invitrogen, ThermoFisher. Elastosil and the curing agent were obtained from Wacker, ABS+ plastic from eSun. Collagen from Advanced Biomatrix and 3,3′‐Dihexyloxacarbocyanine Iodide (DiOC6(3)) from Invitrogen, ThermoFisher.

### Preparation of GAG‐Analog Hydrogel

GAG analogs were prepared using sulfonate‐containing monomers polymerized in the presence of a crosslinking agent, utilizing redox polymerization. Hydrogels were formed at several concentrations (2%, 4%, and 6%) using typical two‐part pre‐gel components. Part (a) contained water, oxone (redox initiator) (0.4 м), and KSPA monomer (6.03 м), while part (b) contained water, AA (redox initiator) (0.15 м), NaAMPS monomer (16.75 м), and PEGDA (crosslinker) at a final concentration of 2%, 4%, 6% w/w), as previously detailed.^[^ ^18^ ^]^ To form a hydrogel, equal volumes of components (a) and (b) were placed on separate catheters and injected simultaneously into the aneurysm cavity, where they were allowed to settle.

### Compression Test

Hydrogels were evaluated for their mechanical properties following predetermined incubation periods (0, 1, 24, 48, 1, 2, and 4 weeks) using a 5544 Tensile Tester Instron (INSTRON, UK), equipped with a 100 N load cell. Briefly, disc‐shaped samples (10 mm in diameter and 5 mm in height) were immersed in PBS for varying durations and subsequently compressed to 80% applied strain at a constant speed of 1 mm min^−1^. Load and displacement were recorded, and the elastic modulus (E) was derived from the linear regression of the stress–strain curve between 10% and 20% strains. Failure stress and strain were defined as the maximum stress and strain, respectively, before the mechanical failure of the hydrogel. Compliance was determined by measuring the strain under pressure ranging from 10.5 to 16 kPa, corresponding to the physiological systolic blood pressure range (80–120 mmHg). For each analysis, five samples were tested, and the mean and standard deviation were calculated.

### Viscosity Measurement of GAG Analogs

The injectability of GAG‐analog pre‐gel solutions was assessed by measuring their viscosity. Pre‐A and Pre‐B solutions, in different crosslinker concentrations (2%, 4%, and 6%), were tested for viscosity using a size 50 and 200 Cannon‐Fenske viscometers.

### Hydration of GAG Analogs

The hydration of polymerized GAG‐analogs hydrogels (2%, 4%, and 6%) was evaluated under confined and unconfined conditions following immersion in PBS at 37 °C for specified time intervals (1, 24, 48 h, 1 and 4 weeks). At each time point, the wet weight and volume of the swollen gel were measured. For unconfined swelling, disc‐shaped samples (10 mm diameter and 5 mm height) were prepared, and their volumes at each time point were calculated by measuring the height and diameter of cylindrical hydrogels. For confined swelling, samples were prepared in 2 mL Eppendorf tubes, and their volume was evaluated by subtracting the complementary volume. The results were expressed as a swelling ratio, and were calculated as follows:

(1) Weightchange%w=(wt−w0)/w0

(2) Volumechange%v=(vt−v0)/v0

where: *w*_0_ and *v*_0_ are the weight and volume of hydrogel at time 0, respectively, *w*_t_ and *v*_t_ are the weight and volume of the hydrogel at time t.

The dry weight was measured after 24 h of lyophilization. Each swelling value was the average of four samples of the same specified time t.

Hydrogel swelling in different liquid media was measured by curing 4% crosslinked hydrogels (final volume 2 mL) within 5 mL Eppendorf tubes and allowing the hydrogels to swell in the presence of PBS, plasma, albumin solution (0.034 gr mL^−1^ in PBS) or human blood (2 mL each) over defined durations (0, 1, 2, 5, 7, and 14 days). The change in hydrogel level was measured daily and the corresponding change in volume was calculated. The results were expressed as the swelling volume ratio (%*v*, Equation (2). The volume was calculated by multiplying the height by the cylinder area.

### ATR‐FTIR Analysis

Attenuated total reflectance–Fourier transform infrared (ATR‐FTIR) spectroscopy was conducted using a Nicolete‐iS50 (Thermo Scientific, USA). The spectra were scanned between 400 and 4000 cm^−1^ with a resolution of 4 cm^−1^.

For the ATR‐FTIR measurements, hydrogels were incubated in Eppendorf tubes for 24 h with fresh human blood, plasma, albumin solution, and PBS at 37 °C. Afterward, the samples were washed with PBS and lyophilized for 48 h, and the top layer was ground into a fine powder for ATR‐FTIR analysis.

### Swelling Pressure

The pressure exerted by the swollen hydrogels (2%, 4%, and 6%) was evaluated under confined and unconfined conditions using a Texas Instrument DHR rheometer (TA Instruments, New Castle, DE, USA) equipped with a parallel plate configuration. For unconfined swelling, disc‐shaped samples (10 mm in diameter and 5 mm in height) were prepared and placed in an open chamber. A 0.1 N pre‐load was applied to the sample to ensure proper confinement. The chamber was filled with PBS to enable sample swelling, and the axial force was monitored while keeping the displacement constant for 8 h, allowing the system to reach the equilibrium swelling. For confined swelling, disc‐shaped samples (5.5 mm in diameter and 5 mm in height) were prepared and placed in a 5.5 mm cylinder chamber designed with pores to enable liquid movement. The swelling pressure was defined as the maximum pressure reached during the test. Confined gel samples were monitored for 6 h until equilibrium swelling was reached.

### Fabrication and Setup of 3D Printed Aneurysm Model

Cerebral aneurysm models with well‐defined geometries (aneurysm aspect ratio AR = 3.2, parent artery curvature radius = 12.5 mm) were constructed using Computer‐Aided Design (CAD) models and were 3D printed using RDG 720 plastic using an Object Eden 500 printer. The molds were then filled with silicone (Dow‐Corning Sylgard184) and mixed with curing agents at a 1:10 mass ratio. The cured molds were placed in an acetone bath overnight to soften the plastic and facilitate its removal from the silicone cast.^[^ ^45^ ^]^

### Aneurysm Model Filling and Flow System

The 3D aneurysm models were filled (up to their neck) using a delivery system comprised of two catheters, each delivering one of the pre‐gel solutions (a total of 300 µL) to a mixing reservoir, using a peristaltic pump at a rate of 0.5 mL min^−1^.

### Swelling in 3D Aneurysm Under Dynamic Conditions

The swelling behavior of GAG analogs (4%) in the presence of three different liquids (PBS, plasma, and human blood) under flow conditions was monitored for 2 weeks. Briefly, pre‐gel solutions (A and B with 4% crosslinker) were inserted simultaneously via wire guides. Following 15 min of gelation, the fluid under investigation was circulated at a rate of 25 mL min^−1^ (100 rpm) using a peristaltic pump. The protrusion outward toward the aneurysm neck was monitored at specified time intervals (0, 1, 2, 3, 7, and 14 days).

Ethical approval was obtained for the experiments with blood samples from human subjects (Rambam Medical Center Institutional Review Board (IRB) RMB‐0413‐21). For blood experiments, whole blood was taken from healthy human volunteers (consent from all participants was obtained prior to the research), and supplied by the Israeli National Blood Service (Rambam Medical Center Institutional Review Board (IRB) RMB‐0413‐21).

### Flow Imaging in Aneurysm Models

10 µm fluorescent carboxylated polystyrene (PS) particles (Thermo‐Fischer) at a concentration of 0.125 µg mL^−1^ were perfused into the Elastosil aneurysm model, which was connected to a perfusion system as previously described.^[^ ^28^, ^29^ ^]^ The flow rate was set to 200 mL min^−1^. Images were taken every 5 s for 10 min to monitor the particles’ pathlines using a Nikon SMZ25 stereo microscope.

### Biocompatibility Assay

To evaluate the biocompatibility of GAG analogs, HUVECs (Catalog # LNW‐2519A, Lonza, Walkersville, MD), passage 3–6, were cultured on top of disc‐shaped hydrogels (4%), fixed, and stained for viability and morphology. Briefly, hydrogels (300 µL) were prepared in 48‐well plates and transferred to 24‐well plates for 24 h of equilibrium in fresh medium prior to cell seeding. The hydrogels were coated with fibronectin (20 µg mL^−1^ in PBS) for 1 h at 37 °C and HUVECs (2 × 10^5^ cells) were seeded on top of the hydrogel discs and in 24‐well plates (positive control). They were further incubated for 1 week using Endothelial Cell Medium (ECM) supplemented with 5% fetal bovine serum (FBS), 1% endothelial cell growth supplement (ECGS), and 1% (v/v) penicillin/streptomycin at 37 °C and 5% CO_2_. The medium was replaced every other day. The viability and morphology of the cells were measured following 24 h and 1 week of incubation. Cells were fixed with 4% formalin for 15 min, washed three times with PBS, permeabilized with 0.5% Triton X‐100 for 10 min at 4 °C, and blocked with 10% FBS. Next, the cells were incubated with phalloidin (1 mg mL^−1^, 1:20 in PBS) for 40 min at RT, and then washed three times with PBS. The cells were further stained with DAPI (1:1000) for 5 min at RT and washed three times with PBS. After 24 h and 1 week in culture, the cells were stained with 24 µм Fluorescein Diacetate and 2 mм Ethidium homodimer I. Images were captured using a Nikon Eclipse *Ti* confocal microscope. To quantify cell viability, the cells were split with trypsin EDTA (LifeGene) and counted daily.

### Hemolysis Assay

The hemolytic activity of GAG analog hydrogels was evaluated by incubating the gels with whole human blood.^[^ ^31^ ^]^ Gels were prepared in 2 mL Eppendorf tubes, rinsed with PBS for 30 min, and then blood was added with PBS in the ratio of 4:5 v/v (test samples TS, n = 3). The mixtures were incubated for 60 min at 37°. After incubation, the samples were centrifuged, and absorbance at 540 nm was measured from the supernatant fluid. Positive control samples (PC) consisted of blood mixed with distilled water; Negative control samples (NC) included PBS (n = 3 for each control). The hemolysis ratio (%HR) was calculated using the following formula:

(3) $$\%HR=100*\frac{TS-NC}{PC-NC}$$

### Platelet's Adhesion Assay

Platelets in fresh human blood were fluorescently stained using 3,3′‐Dihexyloxacarbocyanine Iodide (DiOC6(3)). Then, Platelets rich plasma (PRP) was prepared by centrifugation at 300 g for 20 min.^[^ ^46^ ^]^ GAG analog hydrogels were prepared and placed on 24‐well plates. PRP was added to fully immerse the hydrogels, which were then incubated for 2 h in 37°. Following incubation, the hydrogels were washed with PBS to remove any non‐adherent platelets. Platelet adhesion was assessed using fluorescent microscopy. Positive controls involved detecting platelets adhesion on 10% collagen (VitroCol, Advanced Biomatrix) coated wells.

Ethical approval was obtained for the experiments with blood samples from human subjects (Rambam Medical Center Institutional Review Board (IRB) RMB‐0413‐21). Whole blood was taken from healthy human volunteers, who provided written informed consent, at the Rambam Medical Center (Rambam Medical Center Institutional Review Board (IRB) RMB‐0413‐21). A total of 9 mL of blood was collected from each volunteer into a citrated blood collection tube.

### Statistical Analyses

Statistical analysis was performed using a two‐tailed t‐test and analysis of variance (ANOVA), followed by the Tukey test. All experiments were repeated at least three times, and the data are presented as the mean ± SD. The types of tests and analyses performed in each experiment, and the statistical significance, are depicted in the figure legends. All statistical analyses were conducted using GraphPad Prism 8 (GraphPad Software, Inc., CA, USA). A p‐value < 0.05 was considered statistically significant.

## Conflict of Interest

The authors declare no conflict of interest.

## Supporting information



Supporting Information

## Data Availability

The data that support the findings of this study are available from the corresponding author upon reasonable request.
