# Supplementary material for: Biomimetic Glycosaminoglycan‐Analog Hydrogel for Improved Embolization of Aneurysms: Environment‐Selective Swelling
Source: Adv Healthc Mater. 2025 Apr 7;14(17):2404506. doi: 10.1002/adhm.202404506 (PMC12232164; doi:10.1002/adhm.202404506)
Supplement: Supplementary file 1 — Supporting Information [file ADHM-14-0-s001.pdf]

ADVANCED HEALTHCARE MATERIALS

Supporting Information

for *Adv. Healthcare Mater.*, DOI 10.1002/adhm.202404506

Biomimetic Glycosaminoglycan-Analog Hydrogel for Improved Embolization of Aneurysms: Environment-Selective Swelling

Sarit S. Sivan, Iris Bonshtein, Maria Khoury, Yevgeniy Kreinin, Dmitry Korneyev, Tirosh Mekler, Sumaya Kaiyal, Iris Sonia Weitz and Netanel Korin**

Supplementary Materials

Biomimetic Glycosaminoglycan-Analog Hydrogel for Improved Embolization of Aneurysms: Environment-selective Swelling

Sarit S Sivan[1,*,€], *Iris Bonshtein*[1,2,€], *Maria Khoury*[2], *Yevgeniy Kreinin*[2], *Dmitry Korneyev*[2], *Tirosh Mekler*[2], *Sumaya Kaiyal*[1], *Iris Sonia Weitz*[1], *Netanel Korin*[2,*,€]

[1] *Department of Biotechnology Engineering, Braude College of Engineering, Karmiel 2161002, Israel*

[2] *Department of Biomedical Engineering, Technion-Israel Institute of Technology, Haifa 32000, Israel*

**Corresponding Authors*

E-mail: korin@bm.technion.ac.il; ssivan@braude.ac.il

[€] Equal contribution

This supplementary material includes 2 supplementary figures

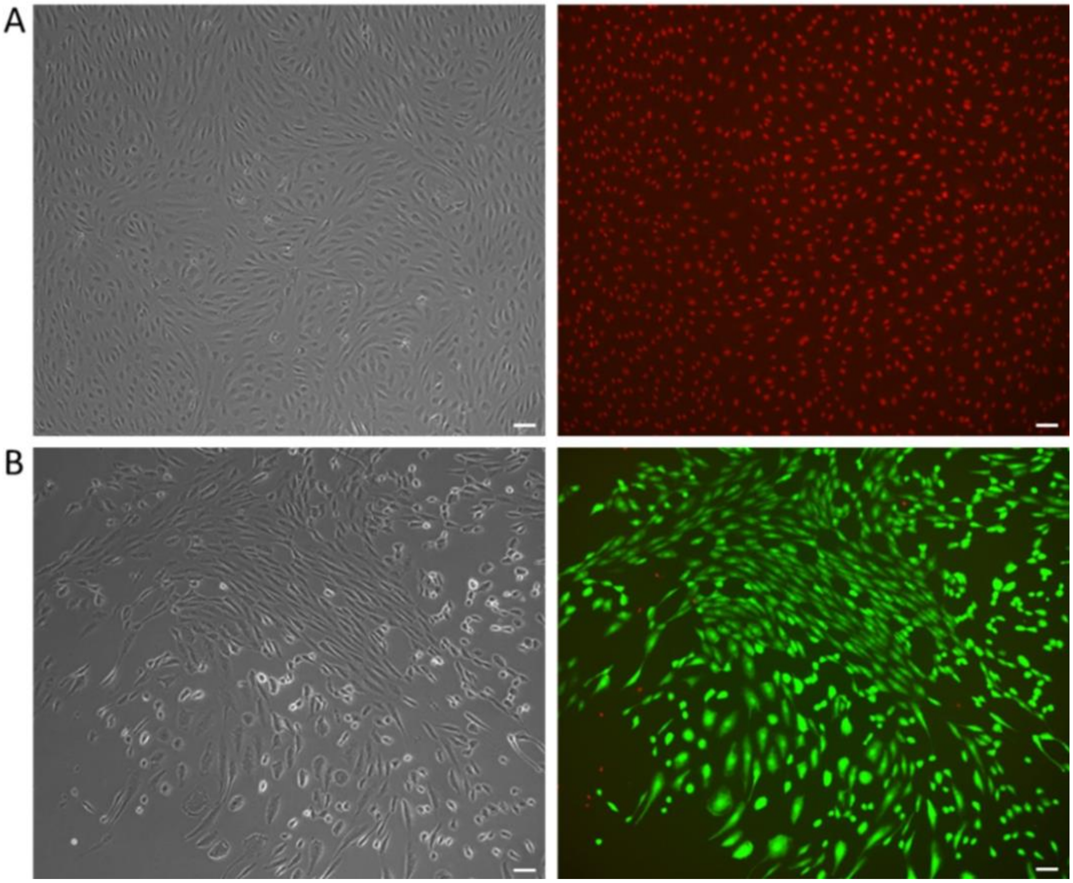

Figure S1: Live/Dead staining of HUVEC cultured on GAG hydrogel on day 7. (A) Methanol was added to the cultured cells for positive control, and all cells were stained with Ethidium Homodimer I (red). **(B)** Stained cells on hydrogel, 7 days following seeding, using the Ethidium Homodimer I and Fluorescein Diacetate. Notably, over 98% of the cells are alive (green). Phase contrast images are also presented for reference. Scale bar: 50 μm.

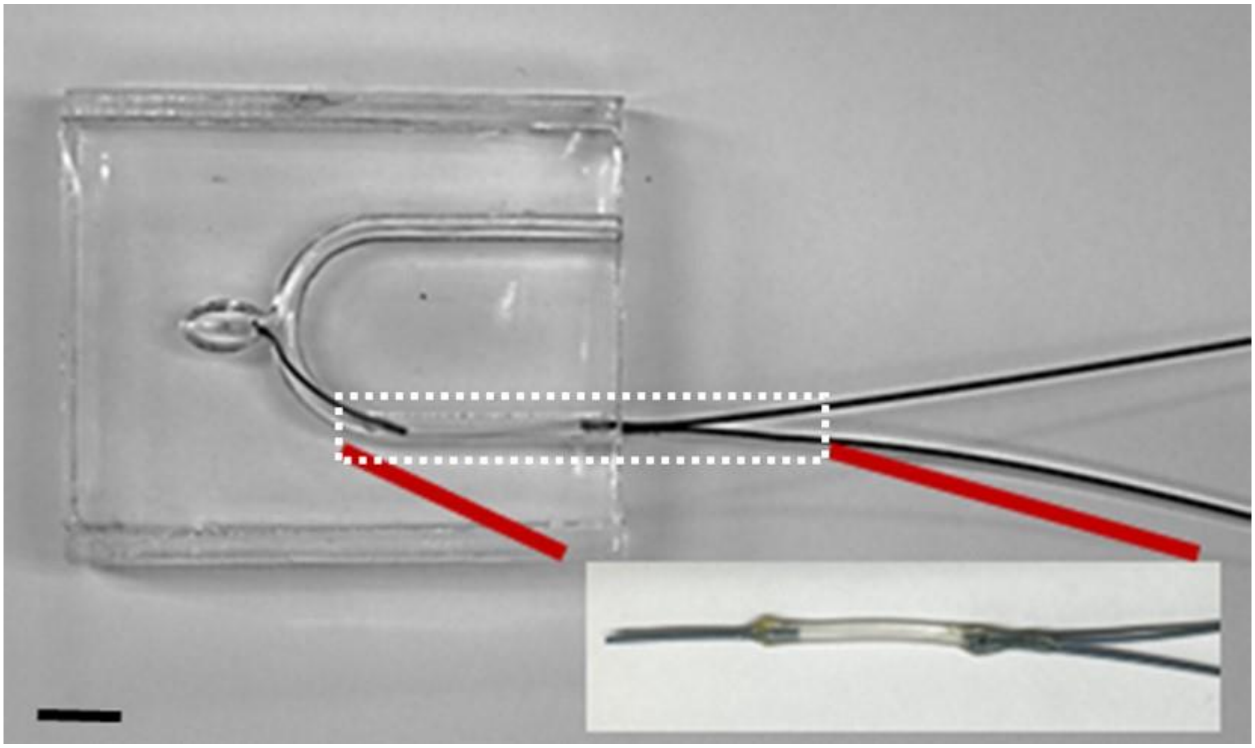

Figure S2: Two-component system used for the injection of GAG analog monomers into the aneurysm model. The two components of the hydrogel are delivered via separate catheters, which merge into a single tubing, where they mix, before filling the aneurysm. The hydrogel mixture is then injected through the tubing using one catheter, where it solidifies and embolizes the aneurysm. Scale bar: 10 mm.
